# Metagenomic Next-Generation Sequencing Is Highly Efficient in Diagnosing Pneumocystis Jirovecii Pneumonia in the Immunocompromised Patients

**DOI:** 10.3389/fmicb.2022.913405

**Published:** 2022-06-17

**Authors:** Dongsheng Wang, Shihua Fang, Xiaowen Hu, Qixia Xu, Xinmin Chu, Xiaodong Mei, Wang Xie

**Affiliations:** ^1^Department of Pulmonary and Critical Care Medicine, Anhui Provincial Hospital, Cheeloo College of Medicine, Shandong University, Jinan, China; ^2^Department of Pulmonary and Critical Care Medicine, The First Affiliated Hospital of USTC, Division of Life Sciences and Medicine, University of Science and Technology of China, Hefei, China; ^3^Wannan Medical College, Wuhu, China; ^4^Department of Clinical Laboratory, The First Affiliated Hospital of USTC, Division of Life Sciences and Medicine, University of Science and Technology of China, Hefei, China

**Keywords:** metagenomics next-generation sequencing (mNGS), pneumocystis jiroveci pneumonia, immunocompromised host, pathogens, diagnosis

## Abstract

**Purposes:**

To explore the value of metagenomic next-generation sequencing (mNGS) in diagnosing pneumocystis jiroveciipneumonia (PJP) in the immunocompromised patients.

**Methods:**

Data of 122 patients with PJP in an immunosuppressed state and 67 non-PJP patients were collected. The diagnostic efficacy of mNGS was compared with the conventional methods, including Gomori methenamine silver (GMS) staining and serum (1,3)-β-D-glucan (BDG). Changes of anti-microbial therapy for patients with PJP based on mNGS results were also reviewed.

**Results:**

The diagnostic sensitivity of mNGS to PJP was higher than that of GMS and BDG (100% vs. 15 and 74.5%, *p* < 0.001). The diagnostic specificity (91.%) was lower than that of GMS (100%), and similar with BDG (89.6%). In addition to P. jirovecii, mNGS revealed co-pathogens like human β-herpesvirus 5, human γ-pesvirus 4, and some other opportunistic pathogens. The reads of mNGS were remarkably higher in BALF than in blood samples. Initial antimicrobial treatment was modified in 89.3% patients based on the mNGS results, and 74 cases (60.7%) were treated with anti-*P.* jirovecii therapy.

**Conclusion:**

mNGS is highly efficient in diagnosing PJP and good at identifying pathogens in mixed infections.

## Introduction

Pneumocystis jirovecii pneumonia (PJP), a severe fungal infection, often occurred in patients with HIV and other immunocompromised individuals (Sepkowitz, 2002). For example, the incidence of PJP in non-HIV immunocompromised population, such as rheumatic disease, organ transplantation, hematologic malignancies, and solid tumors, has been reported increasing (Miller et al., 2006; Kovacs and Masur, 2009). Delayed diagnosis due to relatively poor efficacy of conventional diagnostic methods often results in higher mortality in the patients (Monnet et al., 2008; Li et al., 2014; Bienvenu et al., 2016). For a long time, the diagnostic standard to PJP has been depending on histological and microscopic identification by Gomori methenamine silver (GMS) staining on samples including tissue, bronchoalveolar lavage fluid (BALF), and sputum. However, the diagnostic sensitivity is not satisfactory as the positive rate is rather low (Procop et al., 2004). Serum (Sepkowitz, 2002; Kovacs and Masur, 2009)-β-D-glucan (BDG) is another method widely used but poorly species-specific (Del Corpo et al., 2020). Polymerase chain reaction (PCR) testing is fast and more sensitive than GMS staining. However, the cut value varies in different studies, and there is no uniform value that could be regarded as the positive diagnostic standard yet. Therefore, PCR testing is not widely used in clinical practice (Wilson et al., 2011; Cooley et al., 2014). Apparently, new technology for diagnosis of PJP is urgently needed.

Metagenomic next-generation sequencing (mNGS) is a new diagnostic method that can help detect a large amount of complex and rare pathogens quickly and accurately (Goldberg et al., 2015; Westblade et al., 2016). With the advances in technology, mNGS has been gradually applied to complicated pathogen identifications, especially in the immunocompromised patients (Miao et al., 2018; Xie et al., 2019). Recently, a few case studies have reported that mNGS was more helpful in the diagnosis of PJP than the conventional methods (Miao et al., 2018; Zhang et al., 2021). Recently, in an introspective study, mNGS using blood or BALF samples has been reported useful in diagnosing PJP (Jiang et al., 2021).

However, the strength of most former studies was not strong enough due to the limited number of patients. The diagnostic efficacy of mNGS for PJP remains poorly assessed. In the diagnostic guidelines, the diagnostic significance of mNGS for PJP in non-HIV-infected patients was even not mentioned (Alanio et al., 2016; Fishman and Gans, 2019). Hereby, in this study, we intensively evaluated the efficacy of mNGS in diagnosing PJP in non-HIV-infected patients.

## Materials and Methods

### Study Design

This is a retrospective study conducted at the First Affiliated Hospital of University of Science and Technology of China (Anhui Provincial Hospital). We collected the data of patients from January 2019 to January 2022, who were diagnosed with pulmonary infection and mNGS performed. The patients were then divided into two groups as PJP and non-PJP groups. The clinical diagnosis of PJP was established if a patient met all the following criteria: (1) Immunocompromised conditions, including tumors, hematologic malignancies, rheumatic diseases, systemic use of corticosteroids (dose of 0.3 mg/kg/day of prednisone or equivalent for, continuously, 3 weeks and over), use of immunosuppressive agents (including chemotherapeutic agents for malignancies but not corticosteroids), solid organ transplantation, and hematopoietic stem cell transplantation; etc.; (2) typical PJP clinical manifestations; (3) typical radiologic image changes on chest computed tomography (CT) suggestive of PJP; (4) a positive result of at least one of the following microbiologic tests for *P.* jirovecii: GMS staining or mNGS examination (Fishman and Gans, 2019; Donnelly et al., 2020). For those whose diagnosis was uncertain, the diagnosis was decided by two senior expert pulmonologists (MX and HX) after discussion with the team members based on clinical symptoms, laboratory findings, chest radiology, microbiologic tests, and treatment responses. Immunocompromised was defined by reference to “Treatment of Community-Acquired Pneumonia in Immunocompromised Adults” published in Chest, 2020 (Ramirez et al., 2020). The patient was excluded if he/she met any of the following criteria: (1) age<18 years; (2) HIV infected. This non-interventional study was approved by the Research Ethics Committee at the First Affiliated Hospital of University of Science and Technology of China (No.: 2022-RE-026). All data were de-identified and anonymously processed.

### Sample Processing and DNA Extraction for Metagenomic Next-Generation Sequencing

Volume of 3 ml of blood was drawn from patients, placed in a blood-collection tube and stored at room temperature for 3–5 min before plasma separation and centrifuged at 4,000 rpm for 10 min at 4°C within 8 h of collection. Plasma samples were transferred to new sterile tubes. Approximately, a 1.5–3-ml BALF sample from a patient was collected according to standard procedures. Approximately, a 5-ml microcentrifuge tube, with a 0.6-ml sample and a 250-μl 0.5-mm glass bead, was attached to a horizontal platform on a vortex mixer and agitated vigorously at 2,800–3,200 rpm for 30 min. Then, 7.2-μl lysozyme was added for wall-breaking reaction. Approximately, a.3-ml sample was separated into a new a 1.5-ml microcentrifuge tube. DNA was extracted from 0.3 ml of plasma/BALF using the TIANamp Micro DNA Kit (DP316, TIANGEN BIOTECH, Beijing, China), following the manufacturer’s operational manual. The extracted DNA specimens were used for the construction of DNA libraries (Long et al., 2016).

### Construction of DNA Libraries and Sequencing

Then, DNA libraries were constructed through DNA-fragmentation, end-repair, adapter-ligation, and PCR amplification using MGIEasy Cell-free DNA Library Prep Set (MGI Tech). Agilent 2100 was used for quality control of the DNA libraries. Qualified libraries were pooled; DNA Nanoball (DNB) was made using single-stranded DNA circles and sequenced by MGISEQ-2000/NextSeq 550 platform (Jeon et al., 2014).

### Bioinformatic Analysis

High-quality sequencing data (>10 million) were generated by removing low-quality, short reads (length < 35 bp) (Schmieder and Edwards, 2011), followed by computational subtraction of human host sequences mapped to the human reference genome (hg19) using Burrows-Wheeler Alignment (Li and Durbin, 2010). The remaining data by removal of low-complexity reads were classified by simultaneously aligning with Pathogen Metagenomics Database (PMDB), consisting of bacteria, fungi, viruses, and parasites. The classification reference databases were downloaded from NCBI.^1^ RefSeq contains 4,945 whole genome sequence of viral taxa, 6,350 bacterial genomes or scaffolds, 1,064 fungi related to human infection, and 234 parasites associated with human diseases. The coverage ratio and the depth of each microorganism were calculated using BEDTools (Quinlan and Hall, 2010). The criteria for an infectious pathogen by mNGS were defined as the following: For bacteria, virus, and parasites, if a microbe (a species level) coverage rate scored 10-fold greater than the other microbes of the same type (Langelier et al., 2018); for fungi: a microbe (a species level) coverage rate scored fivfold higher than that of any other fungus because of its low biomass in DNA extraction (Bittinger et al., 2014); for mycobacterium tuberculosis, when at least 1 read was mapped to either the species or genus level due to the difficulty of DNA extraction and low possibility for contamination (Doughty et al., 2014).

### Serum β-D-Glucan Test

Serum BDG was detected by the Kinetic Turbidimetric LAL Kit (Xiamen bioendo technology, China). The result was considered as positive when the BDG value was ≥ 10 pg/ml (normal range: 0–10 pg/ml), according to the manufacturer’s instructions.

### Statistical Analysis

Statistical analysis was performed using SPSS 16.0 software (IBM Corp, Armonk, NY, United States). Continuous variables were presented as medians and interquartile ranges and categorical variables as counts and percentages. Mann–Whitney *U*-test was used for comparing the differences of continuous variables between PJP and non-PJP groups and chi-square test for categorical variables. Sensitivity, specificity, positive predict value (PPV), and negative predict value (NPV) were obtained using the clinical diagnosis as the standard; 95% confidence intervals for these proportions were calculated using Wilson’s method (Tang et al., 2010). Receiver operating characteristic (ROC) curves were constructed for BDG and LDH. The areas under the ROC curves were compared in a non-parametric approach. Statistical significance was defined by *p* < 0.05.

## Results

### Clinical Characteristics

A total of 122 non-HIV-infected PJP patients and 67 non-PJP ones were included in this analysis finally. The median ages in the patients with PJP were younger (55 vs. 67 years, *p*< 0.001). The most common causes of immunosuppressive in the patients with PJP were long-term systemic corticosteroids (79.5%) and immunosuppressive agents administration (77.9%), respectively. Rheumatic diseases (49.2%) ranked the most common disease class in the patients with PJP and followed those solid organ transplantations. In contrast, those immunosuppressive conditions were far less in the non-PJP patients. The most common symptoms in the patients with PJP were fever (66.4%), cough (54.1%), and expectoration (36.1%). One hundred two patients (83.6%) showed hypoxia symptoms, suggesting that most patients were in severe status. Compared with that in the non-PJP group, the white blood cell count (7.6 vs. 9.7 × 10^9^/l, *p* = 0.001) and the neutrophil count (6.5 vs. 7.8 × 10^9^/l, *P* = 0.003) decreased significantly in the patients with PJP. Median serum levels of BDG were significantly increased in the patients with PJP (46.3 vs. 3.8 pg/ml, *p*<0.001). Median serum levels of lactate dehydrogenase (LDH) (598 vs. 707.6 U/l, *p* = 0.744) and C-reactive protein (74.7 vs. 107.3 mg/l, *p* = 0.038) were remarkably increased in both cohorts. In the patients with PJP, the most common images on CT scanning were ground-glass opacity (80.3%), patchy shadowing (68.%), and interstitial patterns (54.1%). Pleural effusion was less seen in the non-PJP patients (13.1% vs. 65.7%, *p*< 0.001) (Table 1).

**TABLE 1:** Clinical characteristics, laboratory examinations, and CT image results of the patients.

Characteristics [median (IQR) or n (%)]	PJP patients (*n* = 122)	Non-PJP patients (*n* = 67)	*P*-value
Age (years)	55 (21–83)	67 (33–87)	<0.001
Male	81 (66.4)	49 (73.1)	0.339
**Clinical symptoms**			
Hypoxia*	102 (83.6)	51 (76.1)	0.21
Fever	81 (66.4)	57 (85.1)	0.006
Cough	66 (54.1)	56 (83.6)	<0.001
Expectoration	44 (36.1)	52 (77.6)	<0.001
Hemoptysis	3 (2.5)	1 (1.5)	0.659
Chest pain	2 (1.6)	0 (0)	0.292
Immunocompromised conditions	122 (100)	22 (32.8)	<0.001
Systemic use of corticosteroids	97 (79.5)	7 (10.4)	<0.001
Use of immunosuppressive agents	95 (77.9)	13 (19.4)	<0.001
Rheumatic diseases	60 (49.2)	6 (9.0)	<0.001
Solid organ transplantation	31 (25.4)	1 (1.5)	<0.001
Hematologic malignancies	17 (13.9)	3 (4.5)	0.043
Solid tumors	13 (10.7)	11 (16.4)	0.305
HSC transplantation	5 (4.1)	0 (0)	0.097
White blood cells (×10^9^/l)	7.6 (0.2–18.0)	9.7 (1.3–26.8)	0.001
Neutrophils (×10^9^/l)	6.5 (0–16.4)	7.8 (1.1–25.4)	0.003
Hemoglobin (g/l)	115 (34–157)	117 (57–159)	0.62
Platelet (×10^9^/l)	183 (1–460)	190 (25–509)	0.132
Serum BDG (pg/l)	46.3 (3.8–1303.2, *n* = 102)	3.8 (0.4–1303.3, *n* = 48)	<0.001
LDH (U/l)	598 (126–3,666, *n* = 71)	707.6 (188–6,165, *n* = 46)	0.744
CRP (mg/l)	74.7 (1.5–383.2, *n* = 116)	107.3 (2.1–311.6, *n* = 64)	0.038
PCT (ng/ml)	0.23 (0.02–58.8, *n* = 92)	0.20 (0.03–38.6, *n* = 58)	0.963
**Chest CT images**			
Ground-glass opacity	98 (80.3)	12 (17.9)	<0.001
Patchy shadowing	83 (68.0)	52 (77.6)	0.163
Interstitial pattern	66 (54.1)	24 (35.8)	0.016
Consolidation	7 (5.7)	10 (14.9)	0.035
Pleural effusion	16 (13.1)	44 (65.7)	<0.001
Cystic changes	9 (7.4)	3 (4.5)	0.434

IQR, interquartile range; HSC, hematopoietic stem cells; PaO_2_, arterial partial pressure of oxygen; FiO_2_, fraction of inspired oxygen; BDG, (1,3)-b-D-glucan; LDH, lactate dehydrogenase; CRP, C-reactive protein; PCT, procalcitonin; CT, computed tomography. *Hypoxia was defined as peripheral oxygen saturation less than 93% or PaO_2_/FiO_2_ less than 300 mmHg while inhaling room air.

### Diagnostic Performance

The diagnostic performance of mNGS was compared with GMS staining and serum BDG detection. In total, mNGS of BALF or/and blood samples was performed in all patients, GMS staining of BALF in 21 patients (20 in PJP and 1 in the non-PJP cohort), and serum BDG in 150 patients (102 in PJP and 48 in the non-PJP cohort). The sensitivity of mNGS was significantly higher than that of GMS staining [100% (95% CI, 97.–100% vs. 15.% (95% CI, 3.2–37.9%), *p* = 0.000] and BDG [100% (95% CI, 97.–100% vs. 74.5% (95% CI, 64.9–82.6%), *p* = 0.000]. mNGS demonstrated a good specificity, similar with that of serum BDG [91.% (95% CI, 81.5–96.6% vs. 89.6% (95% CI, 77.3–96.5%), *p* = 0.793]. Notably, mNGS reached an NPV of 100%, which surpassed either GMS staining or BDG detection (Table 2). It is worth noting that, in the control group, 6 out of 67 patients were also mNGS positive for *P.* jirovecii. However, these cases were judged as *P.* jirovecii colonization instead of infection after careful and intensive discussion among research members.

**TABLE 2:** Diagnostic performance of mNGS, GMS staining, and serum BDG.

Methods		PJP patients (*n* = 122)	Non-PJP patients (*n* = 67)	Sensitivity (95%CI)	Specificity (95%CI)	PPV (95%CI)	NPV (95%CI)
mNGS	+	122	6	100%	91.0%	95.3%	100%
	–	0	61	(97.0–100)	(81.5–96.6)	(90.1–98.3)	(94.1–100)
GMS	+	3	0	15%*	100%	100%	5.6%*
	–	17	1	(3.2–37.9)	(2.5–100)	(29.2–100)	(0.1–27.3)
BDG	+	76	5	74.5%^#^	89.6%	93.8%	62.3%^#^
	–	26	43	(64.9–82.6)	(77.3–96.5)	(86.2–98.0)	(49.8–73.7)

*GMS staining, Gomori methenamine silver staining; BDG, (1,3)-β-D-glucan; serum BDG ≥ 10 ng/l was defined as positive.*

*CI, confidence intervals; PPV, positive predict value; NPV, negative predict value.*

*p < 0.001 when comparing GMS staining with mNGS.

*^#^p < 0.001 when comparing serum BDG with mNGS.*

### Pathogen Characteristics and Treatment Impact

**FIGURE 1:**
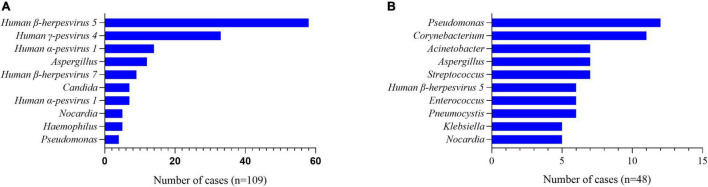
A pathogen spectrum of patients with PJP and non-PJP ones. **(A)** Co-pathogens in the patients with PJP. **(B)** Co-pathogens in the non-PJP patients.

All the patients had BALF or blood samples collected and mNGS performed. Data showed that there were more than one pathogens detected in 89.3% (109/122) of patients with PJP. Viruses, including human β-herpesvirus 5, human γ-pesvirus4, and humanα-pesvirus1, ranked the most common co-pathogens in the patients with PJP (Figure 1A), and, in the non-PJP patients, the most common pathogens were *p*seudomonas, corynebacterium, and acinetobacter (Figure 1B). With the mNGS results, antimicrobial treatment was modified in 89.3% of patients with PJP. Approximately, 45.9% of patients were treated with one or two additional antimicrobial agents. Approximately, 35.2% were added with trimethoprim-sulfamethoxazole (TMP-SMZ), 13.1% were added with caspofungin, and 12.3% were added with TMP-SMZ pluscaspofungin (see Table 3).

**TABLE 3:** Impact of mNGS on antimicrobial treatment for patients with PJP.

Modifications [*n* (%)]	PJP patients (*n* = 122)	Non-PJP patients (*n* = 67)
Antimicrobial treatment change	109 (89.3)	50 (74.6)
Add TMP-SMZ only	43 (35.2)	4 (5.9)
Add caspofungin only	16 (13.1)	0 (0)
Add TMP-SMZ + caspofungin	15 (12.3)	0 (0)
Add agent	56 (45.9)	45 (67.2)
Remove agent	0 (0)	1 (1.5)
No change	13 (10.7)	17 (25.4)

*TMP-SMZ, trimethoprim-sulfamethoxazole; remove agent, the number of antimicrobial agent types reduced after the report of mNGS results; add agent, the number of antimicrobial agent types increased after the report of mNGS results.*

### Power of Metagenomic Next-Generation Sequencing for Bronchoalveolar Lavage Fluid and Blood Samples

**FIGURE 2:**
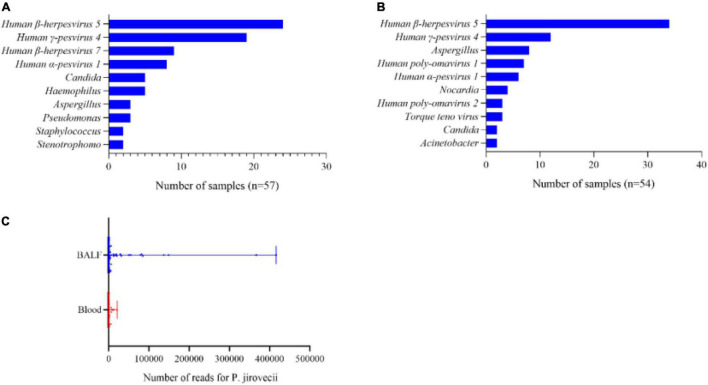
A pathogens spectrum in different samples of the patients with PJP. **(A)** Co-pathogens in BALF samples. **(B)** Co-pathogens in blood samples. **(C)** Reads of *P.* jirovecii in BALF and blood samples. The median reads in BALF were significantly higher than that in the blood samples (990.5 vs. 73, *p*< 0.001).

In total, there were 62 BALF samples and 65 blood samples in the patients with PJP, because 3 of the 122 patients with PJP had their BALF samples and blood samples both collected and mNGS performed. Data showed that there were more than one pathogen detected in 57 BALF samples and 54 blood samples; the most common co-pathogens were human β-herpesvirus 5 and human γ-pesvirus 4 (Figures 2A,B), which were similar in both types of the samples. In addition to the viruses, aspergillus, nocardia, and candida were also detected in the blood samples (Figure 2B). It was note mentioning that the median reads in blood were remarkably lower than that in the BALF samples (73. vs. 990.5, *p*<0.001, Figure 2C).

### Receiver Operating Characteristic Curves-Constructed Analysis

**FIGURE 3:**
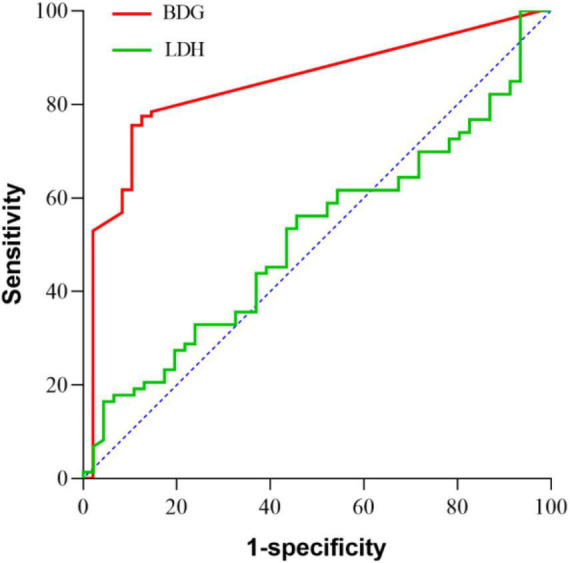
ROC curves for lactate dehydrogenase (LDH), β-D-glucan (BDG). The areas under the curve values are 0.845 (95% CI, 77.8–91.2%, *p* < 0.001) for BDG and 0.482 (95% CI, 37.6–58.8%, *p* = 0.744) for LDH. There were significantly differences in the area under the curve value between BDG and LDH (*p*< 0.001).

To date, the quantitative cutoff or threshold value of BDG and diagnostic value of LDH has not been clearly established in patients with PJP. In this study, we tried to see whether there would be a practical cutoff or threshold value for each of the markers (Tasaka et al., 2007). ROC curves were constructed for BDG and LDH (Figure 3). The area under the curve was 0.845 (95% CI, 77.8–91.2%, *p* < 0.001) for BDG and 0.482 (95% CI, 37.6–58.8%, *p* = 0.744) for LDH. There were significant differences in the area under the curve between BDG and LDH (*p* < 0.001). The cut-off level of BDG was estimated as 8.82 pg/ml. With this cut-off value, the sensitivity and specificity were 76 and 90%. For LDH, as the area under the ROC curve was 0.482 (95% CI, 37.6–58.8%, *p* = 0.744), the cut-off value, sensitivity, and specificity were not calculated out for it was meaningless statistically. A cut-off value could not be suggested either.

## Discussion

In this study, we evaluated the diagnostic performance of mNGS in non-HIV PJP patients. The results showed that mNGS presented an excellent sensitivity and specificity in diagnosing PJP and identifying the co-pathogens in complicated pulmonary infections. Furthermore, the median reads in BALF were significantly higher than that in the peripheral blood, which confirmed that a BALF sample is more suitable than blood for pathogen identification using mNGS.

PJP is a severe fungal infection, which has a high prevalence in the immunocompromised individuals, posing a huge threat to them. The main clinical manifestations were fever, cough, dyspnea, hypoxia, etc. (Gaborit et al., 2019), as found also in our study. Delayed diagnosis and treatment could lead to higher mortality in those patients. Traditionally, the diagnosis of PJP were established on positive GMS staining on induced sputum or BALF and/or a positive PCR assay on a BALF specimen (Alanio et al., 2016). However, the sensitivity of GMS is rather low and far from satisfactory. The sensitivity of GMS in our study was 15% merely. PCR has been reported to be highly sensitive and specific in detecting *P.* jirovecii, but it has not been widely used especially in diagnosing the mixed infection (Mühlethaler et al., 2012). Serum BDG and LDH were both common diagnostic markers reported in PJP. BDG is one of the major components of the fungal cell wall and has been used for the diagnosis of PJP widely. But the quantitative cut-off value has not been clearly established in patients with PJP (Tasaka et al., 2007; Karageorgopoulos et al., 2013). In our study, we observed significant high serum levels of BDG in the patients with PJP. BDG revealed a relatively good sensitivity in diagnosing PJP. The significant differences in the area under the curve value between for BDG and for LDH (*p*< 0.001) suggested that BDG was the more reliable one among the serum markers evaluated for the diagnosis of PJP. Furthermore, in our study, the cut-off value of BDG was 8.82 pg/ml, which was remarkably lower than 80 pg/ml, a cut-off value recommended for diagnosing candida infections (Theel et al., 2013). Of cause, in diagnosing PJP, the BDG detecting results should be interpreted carefully as other fungi infections can also present positive results. The increase of LDH is often seen in patients with PJP. However, the diagnostic significance of LDH elevation is limited in none-HIV patients as LDH could be elevated in diseases like hematologic malignancies, inflammations, and acute lung injury, etc. (Quist and Hill, 1995). In our study, LDH levels increased in the patients with PJP and also non-PJP ones., The results revealed no difference between the two groups, suggesting that LDH was not an efficient differential diagnostic parameter for PJP.

mNGS is an innovative microbiologic diagnostic technology, which detects a vast field of nucleic acids of microorganisms whose sequencing data are included in the database library and allow an unbiased approach to the detection of unknown pathogens (Goldberg et al., 2015; Westblade et al., 2016). Compared to the classical diagnostic methods, mNGS allows sequence-based identification of all potential pathogenic microbes in clinical samples within a comparatively short time. However, although the performance of mNGS is prominent in diagnosing infection disease, its diagnostic efficacy in PJP has not been well evaluated. In our study, the sensitivity of mNGS was 100% in patients with PJP, which was significantly higher than that of GMS staining and serum BDG detection. Furthermore, mNGS presented a specificity of 91.%, similar with that of serum BDG examination. Based on the mNGS results, 35.2% of the patients were given TMP-SMZ, 13.1% of the patients were given caspofungin, 12.3% of the patients were given TMP-SMZ plus caspofungin in our study. These results suggested that mNGS could facilitate rapid and accurate diagnosis and prompt treatment of PJP.

In addition to its excellent performance in diagnosing PJP, mNGS further showed a great advantage in diagnosing mixed infections in the immunocompromised patients. Microbiological culture is the routine method for determining pathogens traditionally. However, a culture result usually reports the dominant bacteria, usually one species only, and cannot present a whole view of the microbes of infection. Far different from culture, mNGS could detect much more pathogens simultaneously. In our study, data showed that 89.3% (109/122) of the patients with PJP had more than one pathogen to be detected. Besides human β-herpesvirus 5 and human γ-pesvirus 4, some opportunistic co-pathogens like aspergillus, nocardia, and candida were also reported. Initial antimicrobial treatment was modified in 89.3% of the patients with PJP. Approximately, 45.9% of them had one or two antimicrobial agents added, demonstrating that mNGS was excellent in searching underlying co-pathogens and of great help for accurate antibiotic treatment in the complicated pulmonary infections.

Considering the possibility of sensitivity difference of mNGS in different samples, we compared the data of mNGS reports from BALF and the blood samples. The results showed that pathogens were similarly large in BALF and the blood samples in the patients **with** PJP (Figures 2A,B). The most common co-pathogens reported by mNGS were human β-herpesvirus 5 and human γ-pesvirus 4, and followed that some opportunistic pathogens. Our data proved that both the BALF and blood samples were suitable for mNGS. In addition, we were impressed that the median reads in the blood samples were significantly lower than that in the BALF samples, which suggests that a false negativity of mNGS result by blood samples might exist. Apparently, BALF samples should be preferred for mNGS when PJP is suspected, and a blood sample could be an alternative when a BALF sample is not obtainable.

There are some weaknesses in this study. Firstly, it is a single-center retrospective study, and intrinsic bias could not be avoided completely; secondly, because of mNGS were performed in the early stage of the patients and only a small proportion of the patients completed GMS staining, which could affect the comparison of sensitivity of mNGS and GMS. Besides, the PCR assay has not been routinely applied to detecting PJP in our hospital. It is a pity that we do not have the firsthand data to compare the diagnostic efficacy of PCR and mNGS. Although mNGS has been proved to be a rather promising technique for diagnosing mixed pathogen infections, the operation procedure time, usually 24–48 h needed, is a bit longer than that of PCR (Mwaigwisya et al., 2015; Qian et al., 2020); thirdly, mNGS could not tell *P.* jirovecii infection from colonization when the read was low. A definite diagnosis could not be established on mNGS alone, considering the possibility of colonization. This will weaken the strength of this study to some extent. Obviously, well-designed prospective studies are worthy of expectation.

## Conclusion

mNGS is a high-efficient diagnostic method for PJP in non-HIV-infected patients. It is highly sensitive and specific and performs well also in detecting co-pathogens in mixed infections. BALF samples should be preferred for mNGS when PJP is suspected. And when BALF samples cannot be obtained, blood samples can be a supplementary alternative.

## Data Availability Statement

The datasets presented in this study can be found in online repositories. The names of the repository/repositories and accession number(s) can be found below: NCBI—PRJNA818432.

## Ethics Statement

The studies involving human participants were reviewed and approved by The First Affiliated Hospital of University of Science and Technology of China. The patients/participants provided their written informed consent to participate in this study.

## Author Contributions

DW and WX designed the study. SF, XH, XC, and QX took responsibility for the integrity of the data and the accuracy of the data analysis. DW wrote the draft of the manuscript. XM revised and edited the manuscript. All authors contributed to writing or reviewing the manuscript, read and approved the final manuscript.

## Conflict of Interest

The authors declare that the research was conducted in the absence of any commercial or financial relationships that could be construed as a potential conflict of interest.

## Publisher’s Note

All claims expressed in this article are solely those of the authors and do not necessarily represent those of their affiliated organizations, or those of the publisher, the editors and the reviewers. Any product that may be evaluated in this article, or claim that may be made by its manufacturer, is not guaranteed or endorsed by the publisher.

## Footnotes

1
https://www.ncbi.nlm.nih.gov/
